# An interaction network of mental disorder proteins in neural stem cells

**DOI:** 10.1038/tp.2017.52

**Published:** 2017-04-04

**Authors:** M J Moen, H H H Adams, J H Brandsma, D H W Dekkers, U Akinci, S Karkampouna, M Quevedo, C E M Kockx, Z Ozgür, W F J van IJcken, J Demmers, R A Poot

**Affiliations:** 1 Department of Cell Biology, Erasmus MC, Rotterdam, The Netherlands; 2 Center for Proteomics, Erasmus MC, Rotterdam, The Netherlands; 3 Center for Biomics, Erasmus MC, Rotterdam, The Netherlands

## Abstract

Mental disorders (MDs) such as intellectual disability (ID), autism spectrum disorders (ASD) and schizophrenia have a strong genetic component. Recently, many gene mutations associated with ID, ASD or schizophrenia have been identified by high-throughput sequencing. A substantial fraction of these mutations are in genes encoding transcriptional regulators. Transcriptional regulators associated with different MDs but acting in the same gene regulatory network provide information on the molecular relation between MDs. Physical interaction between transcriptional regulators is a strong predictor for their cooperation in gene regulation. Here, we biochemically purified transcriptional regulators from neural stem cells, identified their interaction partners by mass spectrometry and assembled a protein interaction network containing 206 proteins, including 68 proteins mutated in MD patients and 52 proteins significantly lacking coding variation in humans. Our network shows molecular connections between established MD proteins and provides a discovery tool for novel MD genes. Network proteins preferentially co-localize on the genome and cooperate in disease-relevant gene regulation. Our results suggest that the observed transcriptional regulators associated with ID, ASD or schizophrenia are part of a transcriptional network in neural stem cells. We find that more severe mutations in network proteins are associated with MDs that include lower intelligence quotient (IQ), suggesting that the level of disruption of a shared transcriptional network correlates with cognitive dysfunction.

## Introduction

Mental disorders (MDs) are categorized by Diagnostic and Statistical Manual of Mental Disorders, Fifth Edition to include neurodevelopmental disorders such as intellectual disability (ID) and autism spectrum disorders (ASD), as well as psychiatric disorders such as schizophrenia.^1^ ID, ASD and schizophrenia were shown to have a strong genetic component.^2, 3, 4^ Recently, many *de novo* gene mutations in patients with these MDs have been identified by high-throughput sequencing approaches.^5, 6, 7, 8, 9^ A substantial fraction of such potentially MD-associated mutations are in genes encoding proteins involved in the functionally related processes of transcriptional regulation or chromatin modification.^5, 6, 7, 10, 11^ For example, out of 40 genes that were recently found to be *de novo* mutated in multiple ASD patients,^6, 7^ which are therefore strong ASD gene candidates, 22 genes encode transcription factors or chromatin modifiers. It is unclear to what extent MD-associated transcriptional regulators act together in the same gene regulatory networks and molecular pathways. Such information is important to appreciate the level of shared etiology within a clinically defined MD. If cooperating transcriptional regulators are associated with different MDs, it may indicate a molecular relation between these MDs. One important predictor of cooperation between transcriptional regulators is their physical interaction. We and others previously showed that physically interacting transcriptional regulators co-localize on the genome, depend on each other for genome-binding and regulate overlapping gene sets,^12, 13, 14, 15^ suggesting their cooperation in gene regulation.

Transcriptional regulators associated with ASD or ID were shown to often have their highest expression early in human brain development, overlapping with stages of neural stem cell (NSC) proliferation and early neuronal differentiation.^16^ This observation suggests that NSC biology is highly relevant for MDs and that NSCs may be a good source to mine for MD-relevant transcriptional networks and regulators. Here we purified transcriptional factors Tcf4, Olig2, Npas3 and Sox2, which are highly expressed in NSCs, identified their co-purifying interaction partners by mass spectrometry and assembled the first transcription factor interaction network in NSCs. Their high expression in NSCs suggested that these transcription factors are relevant for NSC biology and indeed Olig2, Npas3 and Sox2 were shown to be essential for NSC identity.^17, 18, 19^ On an MD-level, *TCF4* haploinsufficiency causes Pitt Hopkins syndrome, which features severe ID, lack of speech, microcephaly and breathing abnormalities.^20, 21^ Several single-nucleotide polymorphisms in the *TCF4* locus are genetic risk factors for developing schizophrenia.^4, 22^ *OLIG2* is triploid in Down syndrome patients. Restoring diploid gene dose for *Olig2* and *Olig1* in a mouse model for Down syndrome showed recovery of the normal balance of inhibitory and excitatory neuronal activity.^23^ *NPAS3* mutations co-segregate with schizophrenia in two families,^24, 25^ but its overall relevance for schizophrenia has remained unclear. *SOX2* mutations cause an Anophthalmia syndrome with associated cognitive defects in about half of the cases.^26, 27^ The resulting interaction network of these four starting transcription factors and their interaction partners contains 206 proteins. We find that the network contains 68 proteins mutated in patients with ID, ASD or schizophrenia, as well as 52 proteins significantly lacking coding variation in the human population. We provide evidence that proteins associated with ID, ASD or schizophrenia can be part of the same transcription network and that within this network, mutation severity correlates with the level of cognitive dysfunction.

## Materials and methods

### Transcription factor purification and interaction partner identification

**Figure 1:**
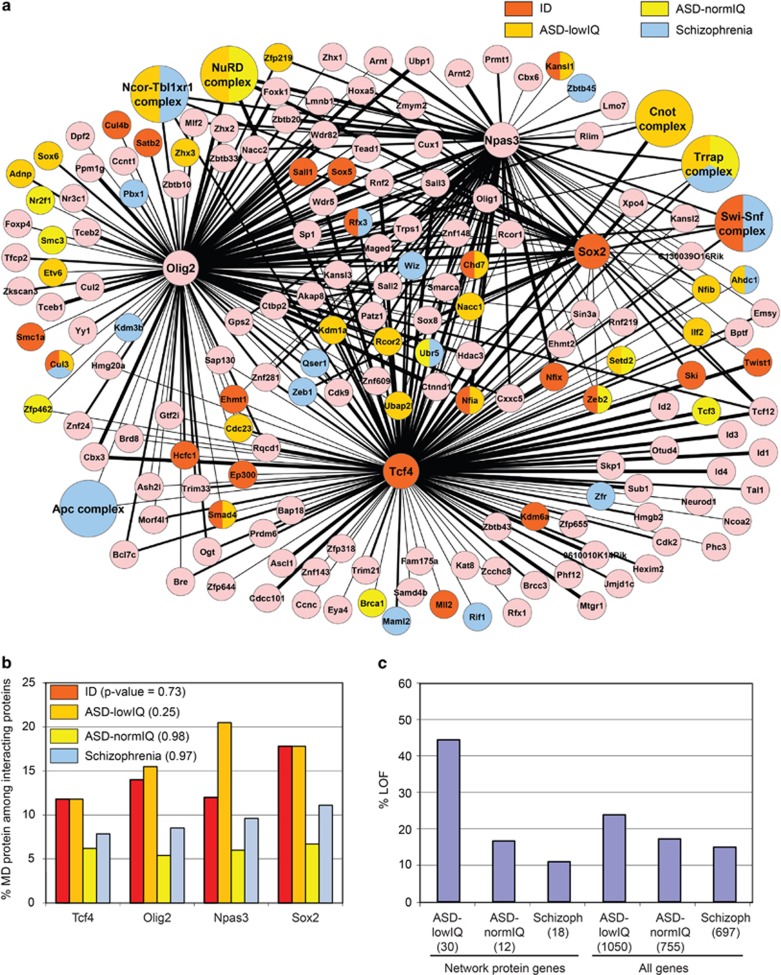
Protein interaction network in neural stem cells. (**a**) Interaction network representing proteins present in two or more purifications of FLAG-tagged Tcf4, Olig2, Npas3 or Sox2 from neural stem cells. Protein complexes are larger circles, thickness of the edges (black lines) gives an indication of protein quantity in samples of FLAG-tagged transcription factor with thickest edges; average emPAI ⩾0.6, medium thick edge; average emPAI <0.6 and ⩾0.2, thin edge; average emPAI <0.2. Red color indicates network protein or protein complex subunit(s) encoded by a known ID gene. Orange, yellow and blue color indicate *de novo* mutation(s) in patients with ASD-lowIQ, ASD-normIQ and schizophrenia, respectively. (**b**) Percentage MD-associated proteins among interaction partners of Tcf4, Olig2, Npas3 and Sox2. MD-categories ID, ASD-lowIQ, ASD-normIQ and schizophrenia are indicated by red, orange, yellow and blue color, respectively. Fisher's exact tests showed no significant differences between the interactomes of Tcf4, Olig2, Npas3 and Sox2 in the percentages of proteins associated with ID, ASD-lowIQ, ASD-normIQ or schizophrenia, *P*-values are indicated. (**c**) Percentage loss-of-function (LOF) mutations in genes mutated in patients with the indicated MD. Network protein genes have the equivalent mouse protein present in the interaction network. Total number of mutations in each category is between brackets. ASD, autism spectrum disorder; ID, intellectual disability; IQ, intelligence quotient; MD, mental disorder.

**Figure 2:**
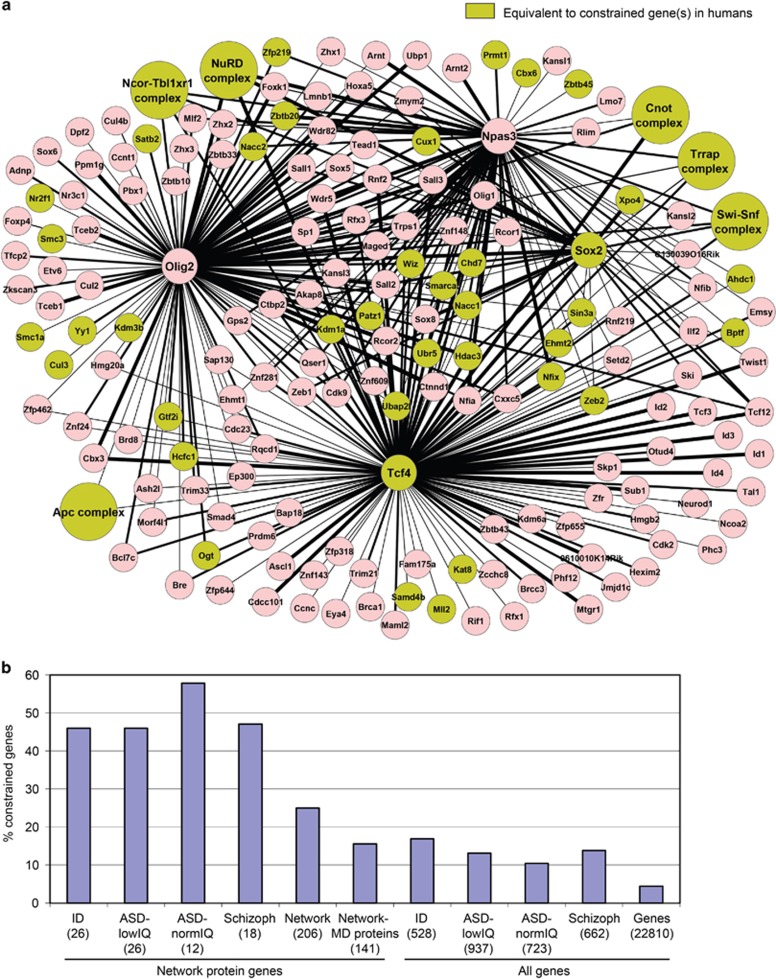
Overlap protein interaction network with constrained human genes. (**a**) Protein interaction network in neural stem cells. Green color indicates overlap of network protein or protein complex subunit(s) with a set of 1003 constrained genes in humans. (**b**) Percentage overlap of the indicated categories with constrained genes. Between brackets is the number of human genes equivalent to network proteins in each category (network protein genes) or total number of genes in each category (all genes). ASD, autism spectrum disorder; ID, intellectual disability; IQ, intelligence quotient; MD, mental disorder.

The NS-5 NSCs were derived from 46C embryonic stem cells^28^ and cultured, as described^29^ and regularly tested for mycoplasma contamination and for authenticity by expressed NSC markers Pax6, Sox2 and Nestin.^15^ The NSCs derived and cultured in this way have the capacity to differentiate into neurons and astrocytes,^29^ and still respond to signals to induce reversible quiescence.^30^ The NSC lines with stable expression of FLAG-V5-tagged Tcf4, Olig2 or Npas3 were created by electroporation with pCAG promoter-driven plasmids containing the appropriate cDNAs and puromycin selection for individual clones with moderate expression of the tagged proteins, as compared with endogenous levels.^15^ FLAG-tagged Tcf4, Olig2 or Npas3 were each purified from 1.5 ml nuclear extract, equivalent to 2 × 10^8^ NSCs, by FLAG-affinity purification, as described.^12, 15^ Two or three biologically independent purifications of each FLAG-tagged protein from separate NSC cultures and control purifications from separate parental NSC cultures were performed by the same experimentor(s). No samples of the above experiments were excluded. Identification of proteins by mass spectrometry was as described.^12^ Peptide spectra of purifications of Tcf4, Olig2, Npas3 and previous purifications of Sox2 (ref. 15) were searched against UniProt release 2012–2011 for protein identification. Interaction partner identification criteria are as described and applied in our previous publications.^12, 15^ In short, a protein is included as interaction partner of a FLAG-tagged transcription factor if present in at least two of its purifications with a Mascot score of 50 or higher and at least threefold enriched by Mascot score over control purifications.^12, 15^ The emPAI score, an estimate of the quantity of the identified protein in the purified protein sample, based on the number of peptide spectra identified by MS, normalized for the number of peptides that theoretically should be identifiable for that protein,^31^ is indicated for each identified protein in each experiment and average emPAI score is used to indicate the thickness of the edges in the interaction network in Figures 1a and 2a. Interaction network graphics were made with Cytoscape.^32^ Large-scale immunoprecipitations from 1 ml of nuclear extracts from NSCs or HEK293T cells were performed as described,^12^ using 10 μg Olig2 antibody (AB9610, Merck, Darmstadt, Germany, RRID:AB_10141047), 10 μg Sox2 antibody (Y-17, sc-17320) or 10 μg Npas3 antibody (HPA002892, Merck, RRID:AB_1079403). Each specific immunoprecipitation was performed once. The resulting western blots were probed with the same antibodies and Ep400 antibody (ab70301, Abcam, Cambridge, UK).

### Chromatin immunoprecipitations

A total 1.5 × 10^8^ NSCs were used per chromatin immunoprecipitation (ChIP). For Olig2 ChIP, NSCs were washed three times with phosphate-buffered saline, crosslinked with 1/10 volume of fresh 11% buffered formaldehyde solution for 12 min, quenched with 1/20 volume of 2.5 m glycine for 5 min, washed with ice-cold phosphate-buffered saline and the cell pellets frozen with N_2_ (l) and resuspended and washed two times in ice-cold cell lysis buffer (10 mm Tris-Cl pH 7.5, 10 mm NaCl, 3 mm MgCl_2_, 0.5% NP40). The cell pellets were resuspended in lysis buffer with 1 mm CaCl_2_ and 4% NP40 and sonicated, as described.^33^ ChIP was performed, as described^34^ using 15 μg of Olig2 antibody (AB9610, Merck, RRID:AB_10141047) or rabbit IgG for the control ChIP. For Tcf4 ChIP, FLAG-V5-Tcf4 expressing NSCs were crosslinked with 2 mm disuccinimidyl glutarate (Thermo Fisher Scientific, Waltham, MA, USA) and 1% formaldehyde, nuclei isolated, chromatin prepared and ChIP performed, as described^33, 35^ with 20 μl V5-antibody agarose beads (Merck). DNA was eluted from the V5-beads, as described.^35^ The NSCs not expressing FLAG-V5-Tcf4 were used as a control. For Npas3 ChIP, NSCs were crosslinked with disuccinimidyl glutarate and formaldehyde and ChIP performed as described^33, 35^ with 15 μg of Npas3 antibody (HPA002892, Merck, RRID:AB_1079403) or rabbit IgG as control, and 60 μl prot-G beads (GE Healthcare, Eindhoven, The Netherlands), without crosslinking the antibody to the beads. Smad4 ChIP was on NSCs crosslinked with disuccinimidyl glutarate and formaldehyde,^33, 35^ with 15 μg of Smad4 antibody (R&D Systems, Minneapolis, MN, USA, AF2097, RRID:AB_355150) or goat IgG and 150 μl prot-G Dynabead solution (Life Technologies, Waltham, MA, USA), without crosslinking the antibody to the beads. Each ChIP-seq for a transcription factor was performed once. ChIP DNA library preparation and ChIP sequencing on Illumina GAII or HiSeq2500 (San Diego, CA, USA) platforms was performed at the Erasmus MC Center for Biomics, as described.^36^

The ChIP-seq data sets were processed and mapped to the NCBIM37.61 (mm9) reference genome, as described.^33^ The published ChIP-seq data sets for Ascl1, Sox2, Brn2, H3K4me1 and H3K27ac in NSCs were retrieved from the Gene Expression Omnibus with accession codes GSE48336, GSE35496, GSE11172 and GSE24164.^37, 38, 39, 40^ The published ChIP-seq data sets for Ep300 and Max in NSCs were retrieved from European Nucleotide Archive with accession codes ERP002084 and ERP004644.^17, 30^ MACS 1.4.2 was used for peak calling and for the generation of binding profiles^41^ using default settings and the corresponding control ChIP as a control data set. The 5000 most significant peaks (genome-wide binding sites) for each transcription factor were used to determine the percentage of overlap between two transcription factors. Two binding sites were considered overlapping if their summits were within 250 bp. The corresponding figures were generated using R. ChIP-seq tracks were generated in the IGV browser.^42^ The ChIP sequencing data are available through the Gene Expression Omnibus (NCBI), accession code GSE70872.

### Gene regulation experiments

**Figure 3:**
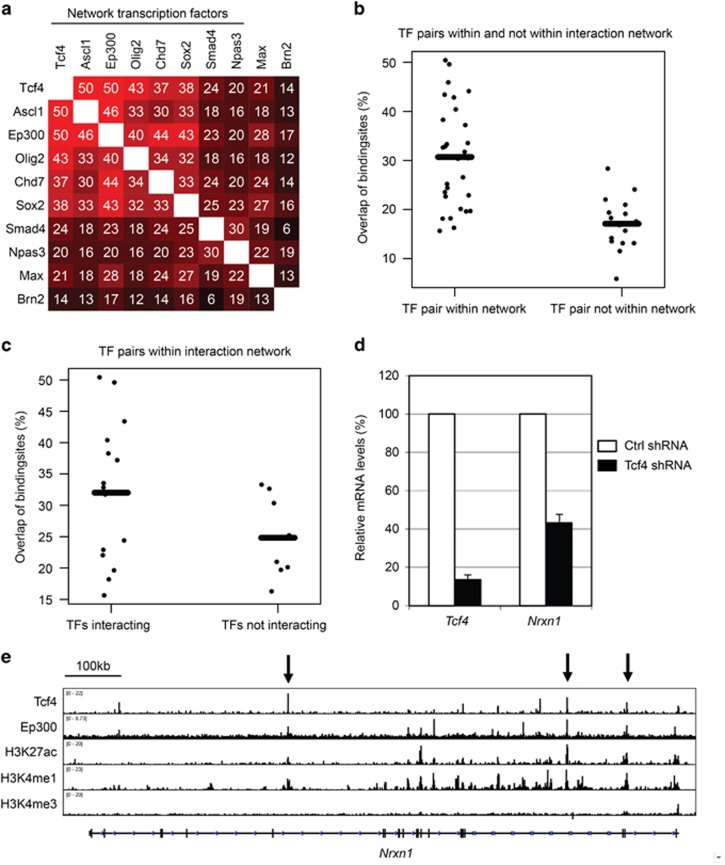
Network transcription factor co-localization on the genome of neural stem cells. (**a**) Percentage overlap of genome-wide binding sites of pairs of transcription factors (TF pairs). Network transcription factors and percentage overlap of TF pair are indicated. (**b**) Transcription factors are both in the network (left) or one is in the network and one is not (right). Each TF pair is indicated by a black dot, average overlap in each category is indicated by black bar. (**c**) Transcription factors are interacting (left) or not interacting (right). Each TF pair is indicated by a black dot, average overlap in each category is indicated by black bar. (**d**) Relative mRNA levels by RT-PCR of indicated genes in NSCs treated with the indicated shRNAs, s.e.m. of three independent experiments is indicated. (**e**) Binding site profile of indicated transcription factors and indicated histone modification profiles at *Nrxn1*. Ep300, H3K4me1 mark enhancers, H3K27ac marks active enhancers, arrows mark transcription factor co-localization. mRNA, messenger RNA; NSC, neural stem cell; shRNA, short hairpin RNA.

**Figure 4:**
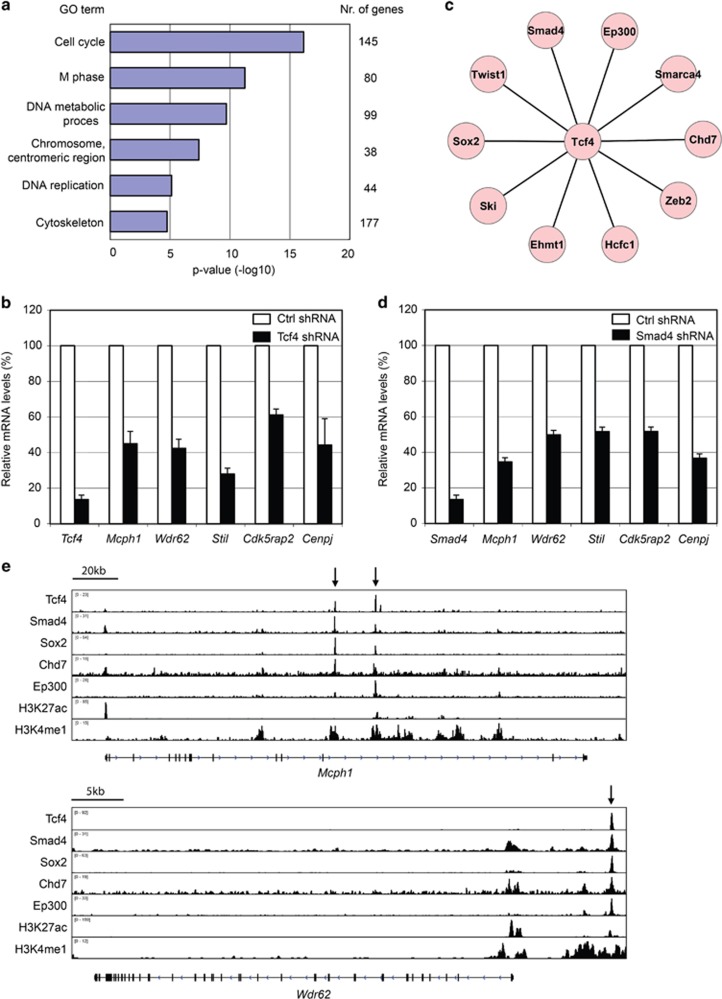
Gene regulation by Tcf4 and its interaction partners. (**a**) Gene Ontology (GO) analysis of putative Tcf4 target genes. Ranking is by Bonferroni-corrected *P*-value. *P*-value (as −log10) and number of Tcf4 target genes in each category is indicated. (**b** and **d**) Regulation of primary microcephaly genes by Tcf4 (**b**) or by Smad4 (**d**), Relative mRNA levels by RT-PCR of indicated genes in NSCs treated with the indicated shRNAs, s.e.m. of three independent experiments is indicated. (**c**) Interaction network of Tcf4 with network transcription factors that are associated with microcephaly. (**e**) Binding site profile of indicated transcription factors and indicated histone modification profiles at *Mcph1* (upper panel) or *Wdr62* (lower panel). *Angpt2*, internal to *Mcph1* and not regulated by Tcf4, is not indicated. Ep300, H3K4me1 mark enhancers, H3K27ac marks active enhancers, arrows mark transcription factor co-localization. mRNA, messenger RNA; NSC, neural stem cell; shRNA, short hairpin RNA.

The pSuper-puro constructs encoding Tcf4 short hairpin RNA (shRNA sequence: 5′-GCACTGCCGACTACAACAG-3′), Tcf4 shRNA2 (5′-GGGTACGGAACTAGTCTTC-3′), Smad4 shRNA (5′-GCTCTGCAGCTCTTGGATG-3′) or Sox2 shRNA^15^ were electroporated into NSCs, as described,^15^ puromycin (2 μg ml^−1^) was added after 18 h and NSCs were collected for analyses at 44 h after electroporation. Three biologically independent electroporations were performed per condition. RNA-seq of untreated NSCs and NSCs transfected with Tcf4 shRNA construct or control shRNA (Dharmacon, Eindhoven, The Netherlands) construct was performed in biologically independent triplicates. poly(A) RNA was isolated using the RNeasy kit (Qiagen, Hilden, Germany), tested for quality with the Bioanalyzer and prepared using the TruSeq RNA sample prep kit v2, as described.^43^ RNA-seq was performed at the Erasmus MC Center for Biomics on a HiSeq2500 sequencer (Illumina) according to manufacturer's instructions. The RNA samples were sequenced for 36 bp. RNA-seq was mapped against mouse reference NCBIM37.67 (mm9) using Tophat50 v2.0.11 with default settings and a segment length of 20. The aligned exon reads were normalized and differential expression was calculated using Bioconductor DESeq2 package in R.^44^ The Tcf4 target genes were defined as having at least a 1.5-fold change in expression (adjusted *P*-value ⩽0.01) upon Tcf4 knockdown, at least one significant Tcf4 binding site (*P*-value ⩽1 × 10^−10^) within 100 kb of its transcription start site and at least 0.1 RPKM expression in untreated NSCs. The RNA sequencing data are available through the Gene Expression Omnibus (NCBI), accession code GSE70872. DAVID (May 2016 gene set update) was used for Gene Ontology analysis^45^ on Tcf4 target genes, Bonferroni-corrected *P*-value was used for ranking Gene Ontology terms. For gene expression analysis, total RNA was isolated from cells using the RNAeasy protocol (Qiagen). cDNA was made with Superscript II reverse transcriptase and RT-PCR was performed on a DNA engine Opticon2/ CFX96 (Bio-Rad, Hercules, CA, USA) and normalized on CalR expression. Each gene expression analysis consisted of three biologically independent experiments. The s.e.m. of these three experiments is shown in Figures 3d, 4b, d and Supplementary Figures 3a–c. No samples were excluded from experiments in this paragraph.

### Data analysis

Known ID genes (528 genes), mutated in five or more ID patients were categorized in Gilissen *et al.,*^5^ Supplementary Table 10. Genes with *de novo* non-synonymous mutations in patients with ASD with lower intelligence quotient (IQ; ⩽90) and patients with ASD with normal IQ (>90) were published in Iossifov *et al.,*^6^ Supplementary Table 7. Likely gene-disrupting mutations^6^ were classified as loss-of-function (LOF) mutations. Genes with LOF mutations in unaffected siblings and genes with only missense (not LOF) mutations in ASD patients and missense mutations in unaffected siblings were removed, as these are less likely to be relevant for ASD pathology, resulting in a list of 1584 genes. Males with ASD and lower IQ (⩽90) and females with ASD (which nearly always have lower IQ)^6, 46^ were classified as ASD with lower IQ (ASD-lowIQ). Males with ASD and normal IQ (>90) were classified as ASD-normIQ. Genes with *de novo* LOF and missense mutations in schizophrenia patients (662 genes, Supplementary Table 10) were categorized from literature.^8, 9, 47, 48^ Frameshift mutations, nonsense mutations and splice-site mutations (within two nucleotides) from the splice donor site or splice acceptor site^9^ were taken as LOF mutations. LOF mutations were calculated as a percentage of all *de novo* coding mutations (LOF+missense) in patients with ASD-lowIQ, ASD-normIQ or schizophrenia, either in genes with the equivalent mouse protein in the interaction network (network protein genes) or in the total mutation data sets (all genes). For known ID genes with the equivalent mouse protein in the interaction network, the type of mutation in the majority of patients was assessed per gene and assigned in Table 1 as LOF or missense.

Enrichment in the network of ID genes equivalent to network proteins was calculated over the expected value in case of a random overlap, which was corrected for protein length and expression in our NSCs by using average protein length from Ensembl genes of network proteins over NSC-expressed genes. Protein length was calculated from ensembl GRCm38 by counting the number of amino acids for each protein. A gene was regarded as expressed in our NSCs, if its expression was equal or above that of *Zeb1* (0.127 RPKM in our RNA-seq data set), which is the network protein with the lowest messenger RNA (mRNA) expression in our NSCs. Genes significantly devoid of coding variants in the human population (1003 genes), also called constrained genes, were reported.^49^ Enrichment in the network of network proteins encoding constrained genes was calculated over the expected value in case of a random overlap, which was corrected for expression in our NSCs. Network-enrichment *P*-values for network proteins encoding known ID genes or constrained genes are obtained from two-sided binomial tests on the observed and expected values. Enrichments and enrichment *P*-values of *de novo* non-synonymous mutations in ASD patients, their healthy siblings or schizophrenia patients in human genes equivalent to network proteins were calculated by dnenrich,^9^ corrected for gene length, sequence context and expression of the mouse homolog in our neural stem cells. Equivalent human genes of network proteins were provided to the dnenrich program as ‘Gene set' and human equivalents of genes expressed in mouse NSCs were provided as ‘Background list'.

Significance of differences in the percentages of proteins associated with ID, ASD-lowIQ, ASD-normIQ or SZ in the interactomes of Tcf4, Olig2, Npas3 and Sox2 and significance of differences in percentages LOF between network protein mutations in patients with ASD-lowIQ, ASD-normIQ or schizophrenia were calculated using Fisher's exact test, as some of the expected counts in the contingency tables were below 5. To determine whether the percentages LOF in network protein mutations in patients with ASD-lowIQ, ASD-normIQ or schizophrenia were differently distributed than equivalent percentages LOF in the total mutation sets, we performed a permutation test by sampling 10 000 random subsets of 60 mutations; the number of mutations in patients with ASD or schizophrenia, identified in our interaction network. The resulting permutation *P*-value was calculated by dividing the number of observed *P*-values that were more significant than the *P*-value (0.002) for our interaction network (34 observations) by the total number of observations (10 000). Significance of differences in LOF percentages in mutations in ASD-lowIQ, ASD-normIQ and schizophrenia categories between network proteins and the total mutation data sets were calculated by Fisher's exact test.

Thirteen human primary microcephaly genes (*MCPH1*, *WDR62*, *ASPM*, *CASC5*, *CENPJ*, *CENPE*, *CDK5RAP1*, *CEP135*, *CEP152*, *STIL*, *CDK6*, *ZNF533*, *PHC1*) are known,^50, 51^ which were overlapped with Tcf4 target genes (see below). Microcephaly genes were retrieved from the Online Mendelian Inheritance in Man (http://www.omim.org) database by scoring for genes in which mutations cause human monogenic conditions or syndromes that include microcephaly.

## Results

### Identification of a protein interaction network in NSCs

We recently improved the FLAG-tag affinity protocol to purify transcription factors and their interacting proteins with high efficiency and low background.^12, 15^ The accuracy of interaction partner identification by this protocol was extensively validated by independent immunoprecipitations.^12, 15^ Importantly, many identified interactions were shown by us (Supplementary Table 1) and others (Supplementary Table 2) to be biologically relevant and uncover novel functions of the target protein or provide insight into the molecular cause of malformations associated with human syndromes.^15^ Here, we applied this protocol to purify transcription factors Tcf4, Olig2 and Npas3 from mouse NSCs. Tcf4, Olig2 and Npas3 have relatively high endogenous expression levels, as compared with the median expression level in our NSCs, determined by RNA-seq (Supplementary Table 3). NSC lines with stable expression of FLAG-tagged Tcf4, Olig2 or Npas3 (Supplementary Figure 1) were grown to large scale and two or three independent purifications of the FLAG-tagged proteins were performed. Interacting proteins, identified by mass spectrometry, present in at least two purifications of the target protein were included (Supplementary Tables 4, see the ‘Materials and methods' section for inclusion criteria) and combined with the interaction partners of previously purified Sox2 (ref. 15; Supplementary Table 7). This resulted in a protein interaction network of 206 proteins and 401 protein–protein interactions (Figure 1a, Supplementary Tables 8 and 9), including 13 protein–protein interactions that were previously shown to be biologically relevant (Supplementary Table 9). The interaction network contains multiple chromatin modifying complexes, such as NuRD, SWI-SNF and Ncor, and transcription factors such as Rfx3 and Sall3 that interact with all four purified transcription factors (Figure 1a). However, other identified interaction partners were found to be specific for one purified transcription factor, such as Ascl1, Neurod1, Kdm6a (Tcf4), Satb2, Yy1, Adnp (Olig2), Arnt2, Lmo7 (Npas3) and Xpo4 (Sox2) (Figure 1a). We confirmed by immunoprecipitations the interaction of endogenous Olig2 with Sox2 (Supplementary Figure 2a) and the interaction of endogenous Npas3 with Sox2 and Ep400 (Supplementary Figure 2b).

### The interaction network is enriched for proteins mutated in patients with ID, ASD or schizophrenia and proteins significantly lacking coding variation in humans

We investigated whether the proteins in our interaction network are associated with ID, ASD or schizophrenia. To our knowledge, the most extensive and best validated list of genes associated with (often syndromic) ID is a list of 528 ‘known ID genes',^5^ which are mutated in at least five ID patients. We find 26 network proteins encoded by ID genes (Figure 1a, Table 1). Taking into account only genes expressed in our NSCs and correcting for protein length, a random overlap would give 9.6 proteins encoded by ID genes in the network. We therefore find a 2.7-fold enrichment of proteins encoded by ID genes in the network, over the expected value (enrichment *P*-value 7.2 × 10^−6^).

To probe for genes in which mutations are unambiguously associated with (non-syndromic) ASD or schizophrenia is a difficult task as few such genes are currently identified. Therefore, as a source for candidate ASD genes, we used a recent large exome sequencing study in 2500 ASD families,^6^ which has the additional benefit to separate ASD patients by low IQ (⩽90, ASD-lowIQ) or normal IQ (>90, ASD-normIQ). The study identified *de novo* LOF (frameshift, nonsense, splice-site) mutations or missense mutations in 1584 genes specifically in patients with ASD-lowIQ or ASD-normIQ.^6^ We find that the interaction network contains 38 proteins encoded by such putative ASD-associated genes with, in total, 42 mutations (Figure 1a, Table 1). A random overlap with the network, corrected by dnenrich^9^ for gene length, sequence context and expression in neural stem cells, would expect 31.5 of such mutations in the network (enrichment *P*-value 0.04). Mutations in unaffected siblings (24 mutations observed in the network, 22.5 expected, *P*-value 0.40) are not enriched in the network. To overlap with schizophrenia candidate genes, we merged mutation data from four exome sequencing studies^8, 9, 47, 48^ and curated a list of 662 genes with LOF or missense *de novo* mutations in schizophrenia patients (Supplementary Table 10). We find 18 network proteins encoded by putative schizophrenia-associated genes with 18 mutations (Figure 1a, Table 1), where the corrected expectation would be an overlap of 12.2 mutations (enrichment *P*-value 0.07). In total, the network contains 68 proteins mutated in patients with ID, ASD and/or schizophrenia and these 68 MD-associated proteins have 260 interactions with other proteins in the network (Figure 1a, Table 1, Supplementary Tables 4). We identified 47 interactions between ID-associated network proteins and network proteins mutated in patients with ASD or schizophrenia (Figure 1a, Supplementary Table 11). We do not find significant differences in the percentages of proteins associated with ID, ASD or schizophrenia between the interactomes of starting transcription factors Tcf4, Olig2, Npas3, Sox2 (*P*-value >0.2 for all MD categories, Figure 1b). Together, our results show that the network is enriched for MD-associated proteins and that the different categories of MD-associated proteins appear homogenously distributed in the network.

To have an indication of the potential of our interaction network to contain yet undiscovered MD proteins, we overlapped our network with a recently reported list of 1003 genes that are significantly devoid of missense variants in the human population^49^ (Figure 2a). These genes are likely to be evolutionarily constrained and intolerant to mutation. Indeed, highly constrained genes were found to be far more often associated with dominant Mendelian disease than genes with an average constraint.^49^ Mutations in the above set of 1003 constrained genes were found to be overrepresented in ASD patients.^49^ Accordingly, we find that genes mutated in patients with ID, ASD-lowIQ, ASD-normIQ or schizophrenia are between two- and fourfold enriched for this set of constrained genes (Figure 2b). We find that a quarter (52 proteins) of the proteins in our interaction network overlap with the set of constrained genes (Figures 2a and b, Supplementary Table 8), a 4.3-fold enrichment over an NSC expression-corrected random expectation (12.2 proteins, enrichment *P*-value 1.9 × 10^−18^). Proteins encoded by constrained genes are still enriched in the network after removal of MD-associated proteins (21 observed, 7.9 expected, *P*-value 6.7 × 10^−5^, Figure 2b). Remarkably, in each of the four MD categories, around 50% of the network proteins with mutations in MD patients are encoded by constrained genes (Figure 2b, Supplementary Table 8). The observed enrichments suggest that network proteins, in particular, those encoded by constrained genes, would be good candidates for mutation screening in patients to identify novel MD genes.

### Mutation severity in network proteins correlates with cognition levels in the associated MD

Cognitive ability is more affected in patients with ASD-lowIQ than in patients with ASD-normIQ or schizophrenia. We were interested whether mutation severity in network proteins would correlate with cognitive ability, with LOF mutations, on average, affecting gene function more severely than missense mutations. We used mutations from our data sets on patients with ASD-lowIQ, ASD-normIQ and schizophrenia, which are three comparable *de novo* mutation data sets derived from whole-exome sequencing of the respective patients and their parents. We find that 43% of the network protein mutations in patients with ASD-lowIQ are LOF mutations, which is 2.5-fold higher than in network protein mutations in ASD-normIQ patients (17% LOF) and nearly fourfold higher than in network protein mutations in schizophrenia patients (11% LOF; Figure 1c, Table 1). The differences in %LOF in network protein mutations between the three MD categories are significant (*P*-value=0.002). %LOF in the total mutation data sets were also different between ASD-lowIQ (24% LOF), ASD-normIQ (18% LOF) and schizophrenia (15% LOF, Figure 1c) but the fold differences were less distinct. Indeed, we find that percentages LOF in our network are differently distributed than percentages LOF in equally sized subsets from the total mutation sets (*P*=0.0034, 10 000 permutations). The difference in mutation distribution between the network and the total data sets is mostly due to the different LOF percentages in ASD-lowIQ (Figure 1c). We find that LOF mutations are significantly overrepresented in the ASD-lowIQ category in our network, as compared with the total mutation set (odds ratio 4.2, *P*-value 1.5 × 10^−4^), whereas this is not the case for ASD-normIQ (odds ratio 0.94, *P*-value 1) and schizophrenia (odds ratio 0.72, *P*-value 1).

One explanation for the exaggerated fold differences in percentages LOF between MD categories within the network could be that mutations in network protein genes more likely contribute to the MD, as supported by the high overlap of mutated network proteins with constrained genes (Figure 2b), a category of genes in which mutations more often cause disease.^49^ In this scenario, the total sets of mutations in the different MD categories (Figure 1c) would contain higher frequencies of non-contributing mutations, which by definition will not have a severity bias between the different MD categories. We also find a mutation bias in network proteins with multiple *de novo* mutations, associated with different MDs; six network proteins have missense mutations in patients with ASD-normIQ or schizophrenia and LOF mutations in patients with ID or ASD-lowIQ, whereas the opposite pattern is not observed (Table 1). Together, this suggests that particularly in network proteins, the severity of mutations increases in MDs with a low IQ.

### Network transcription factors preferentially co-localize on the genome and cooperate in disease-relevant gene regulation

Having identified an interaction network containing MD-related proteins, we investigated whether network proteins preferentially overlap in their genome-wide binding sites, as a proxy for their cooperation in gene regulation.^13, 52^ We determined the genome-wide binding sites in NSCs for network transcription factors Tcf4, Olig2, Smad4 and Npas3 by ChIP-seq and added published data for Chd7, Sox2, Ascl1 and Ep300. We also included published data on Brn2 and Max, two transcription factors with NSC expression levels similar to the tested network transcription factors (Supplementary Table 3) but not part of our interaction network. We find that overlaps in the top 5000 binding sites between transcription factors within the network are on average higher than with Max or Brn2 (Figures 3a and b). Overlaps above 30% are only observed within the network (Figures 3a and b) and overlaps above 35% are only observed between network transcription factors that interact with each other (Figures 3a, c and 1a).

We subsequently explored whether the interaction network can provide gene regulatory explanations for disease overlap. We performed RNAi-mediated knockdown of Tcf4 in NSCs, shortly followed by RNA sequencing. Identified Tcf4 target genes, which are misregulated on Tcf4 knockdown and bound by Tcf4 (Supplementary Table 12), include 71 ID genes, 210 genes *de novo* mutated in ASD patients and 85 genes *de novo* mutated in schizophrenia patients and include well-known MD genes *Foxp2*, *Shank3* and *Syngap1* (Supplementary Table 13). We find that Tcf4 maintains the expression of *Nrxn1* and binds to several active enhancers in the *Nrxn1* gene (Figures 3d and e, Supplementary Table 12). Patients with compound heterozygous mutations in *NRXN1* suffer from Pitt Hopkins-like syndrome.^53^ Regulation of *Nrxn1* by Tcf4 provides a mechanistic explanation for the strong phenotypic overlap in patients with mutations in any of these two genes. Tcf4 and its interactor Sox2 regulate and co-localize on ID genes *Gpr56*, *Tgfbr2* and *Gli2* (Supplementary Figures 3a–d). Disruption of the regulation of *GPR56* by RFX proteins causes cerebral cortex patterning defects and ID.^54^ Rfx proteins interact with Tcf4 and Sox2 (Figure 1a), suggesting their cooperative regulation of *Gpr56*.

Congenital or acquired microcephaly occurs in the majority of patients with Pitt Hopkins syndrome, caused by *TCF4* mutations.^55^ We found that target genes activated by Tcf4 have the highest Gene Ontology term enrichments for gene categories such as *Cell cycle*, *M-phase*, *Chromosome centromeric region* and *Cell division* (Figure 4a), related categories that are often affected in primary microcephaly.^50^ Indeed, Tcf4-activated target genes include 6 of the 13 known primary microcephaly genes (Supplementary Table 13, Figure 4b) and we find that Tcf4 also maintains the expression of primary microcephaly genes *Cenpj* and *Cdk5rap2* (Figure 4b). Moreover, Tcf4 protein interacts with 10 transcriptional regulators associated with microcephaly, including Smad4 (Figures 1a and 4c). Smad4, like Tcf4, regulates primary microcephaly genes (Figure 4d). Tcf4 binds together with microcephaly-associated transcription factors Smad4, Sox2, Chd7 and Ep300 to active enhancers at primary microcephaly genes *Mcph1* and *Wdr62* (Figure 4e). In conclusion, we identified a regulatory network in NSCs related to microcephaly, which may explain the association of this condition with the participating proteins.

## Discussion

### A transcription factor interaction network in NSCs enriched for MD-associated proteins

Here we believe we describe the first transcription factor interaction network in a neural system. We find that the network is enriched for proteins associated with ID, ASD or schizophrenia. Accordingly, we provide a description of the molecular environment of such proteins, often for the first time, in a cell type highly relevant for neurodevelopment and its diseases. We carried out our studies in mouse NSCs, as the necessary scale of our proteomics and ChIP-seq experiments would be difficult to perform using human NSCs. Nevertheless, a recent comprehensive comparison of transcriptional networks and transcription factor target genes in mouse and human shows high inter-species conservation,^56^ making our work relevant for the human situation.

The enrichment in the network was highest for a set of established ID proteins.^5^ Enrichments were lower for ASD-associated and schizophrenia-associated mutations, which is possibly due to their origin from sets of *de novo* mutations in ASD or schizophrenia patients, where causality is less certain. Protein mutations in these sets that do not contribute to disease would not be enriched in the network but would increase the expected mutation score, reducing the observed enrichments. The network is highly enriched for proteins encoded by genes significantly lacking coding variation in the human population, a set of ~1000 constrained genes that is more frequently associated with disease, including ASD.^49^ The enrichment for constrained genes would suggest that the network has an above-average content of yet-to-be-discovered MD proteins. Indeed, recently three additional bonafide ID genes were discovered, *RLIM*, *ZBTB20* and *JMJD1C*,^57, 58, 59, 60^ that are encoding network proteins and can be added to 26 ID proteins in the network from the overlap with the 2014 ID gene list.^5^

### Genome co-localization and cooperation in gene regulation by proteins in the network

Transcription factors that interact are more likely to cooperate in gene regulation. Another proxy for cooperation in gene regulation is co-localization on the genome.^13, 52^ For example, we previously showed that Sox2 and its interaction partner Chd7 have a high binding-site overlap on the genome and indeed Sox2 and Chd7 have a large overlap (50%) in regulated genes.^15^ Accordingly, to have an additional indication that network proteins preferentially cooperate with each other in gene regulation, we showed that eight network transcription factors have, on average, more overlap in binding sites with each other than with two transcription factors, Brn2 and Max, that are expressed in NSCs but are not part of the network. Tcf4 binds with a number of its interaction partners to primary microcephaly genes and (at least) Tcf4 and Smad4 also regulate such genes, providing an explanation for their shared microcephaly phenotype. In conclusion, our interaction network shows features of a transcriptional network, where proteins can cooperate to regulate disease-relevant genes.

### Mutation severity in network proteins correlates with IQ levels in the associated MD

We wondered why in our interaction network some mutations occur in patients with ASD-lowIQ, whereas others occur in patients with ASD-normIQ or schizophrenia, MDs without obligatory loss of IQ. One hypothesis would be that severe mutations disrupt the network more and have a worse outcome for IQ levels. Mutation severity within a network of interacting proteins has not been analyzed yet in relation to cognition levels. We find that %LOF rates in our interaction network of transcriptional regulators increase several fold from schizophrenia or ASD-normIQ, two MDs without IQ loss, to ASD-lowIQ, a significantly different distribution than in the total mutation data sets from which they originate (Figure 1c). In addition, in network proteins with multiple mutations across different MDs, mutation severity follows MD severity. A recent data analysis^9^ using *de novo* mutations across all genes in severe ID patients (IQ<50),^61, 62^ ASD patients^63, 64^ and schizophrenia patients^9^ showed an increase in %LOF from 15% in schizophrenia patients, to 17% in ASD patients, to 24% in severe ID patients. These LOF percentages for ASD and schizophrenia do not deviate much from those in the total mutation data sets that we used, suggesting that also here %LOF increase with lower cognition is more modest than in the network. Interestingly, %LOF for network proteins associated with ASD-lowIQ is nearly twice the %LOF value in the set of *de novo* mutations in severe ID patients,^61, 62^ a group with a more severe cognitive deficit. One has to take here into account that in these studies severe ID patients were counter selected for the co-occurrence of congenital anomalies,^61, 62^ which likely impacts on the set of detected *de novo* gene mutations and %LOF. We argued above that the exaggerated increase of average mutation severity with lower IQ in the network may be caused by mutations often being in network proteins encoded by constrained genes and therefore more likely to be causal. This would imply that network mutations and their LOF percentages provide a better reflection of the real mutation spectra associated with the different MD categories than can currently be obtained from the total mutation data sets.

There are a number of limitations to our study. We investigated only the 68 proteins mutated in MD patients present in our network, whereas it is estimated that mutations in several hundreds of proteins may contribute to mental disorders.^65^ Therefore, our protein set represents only a fraction of the estimated number of MD genes. For ASD and schizophrenia, most of the included proteins are not necessarily causally linked to disease, as they were observed in a single patient. Although most MD-mutated transcriptional regulators are highest expressed in NSCs, we cannot exclude that they are relevant for disease in other stages of brain development, such as neuronal maturation, where our network may be less relevant.

Our data are consistent with a scenario where the level of *in vivo* disruption of a shared transcriptional network correlates with the level of cognitive dysfunction in the associated MDs. Our interaction network contains only a fraction of the total number of transcriptional regulators believed to be associated with ID, ASD or schizophrenia, but there is no *a priori* reason to assume that its principles would not apply to a larger network of MD-related transcriptional regulators. A shared underlying transcriptional network is in line with the significant comorbidity observed between ID, ASD and schizophrenia.^66, 67^

## Figures and Tables

**Table 1:** Network proteins with mutations in the equivalent human gene in patients with a mental disorder

*Human homolog* *of network protein*	*ID*	*ASD-lowIQ*	*ASD-normIQ*	*Schizophrenia*	*Constrained* *gene*	*Severity mutation* *vs* *IQ in MD*
TCF4	LOF				X	
ZEB2	LOF		Missense		X	**>**
SMC1A	Missense				X	
SATB2	LOF				X	
SOX2	LOF				X	
CHD7	LOF	Missense			X	
RFX3	LOF			Missense		**>**
HCFC1	Missense				X	
CUL3	LOF	LOF		Missense	X	**>**
SALL1	LOF					
MLL2	LOF				X	
EHMT1	LOF					
SOX5	LOF					
KANSL1	LOF	Missense				
EP300	LOF			Missense		**>**
TWIST1	LOF					
KDM6A	LOF					
NFIA	LOF	LOF				
SKI	Missense					
NFIX	LOF				X	
SMAD4	Missense	Missense				
ARID1A	LOF					
SMARCE1	Missense					
SMARCB1	Missense				X	
SMARCA4	Missense				X	
CUL4B	LOF					
ADNP		LOF 2 ×				
AHDC1		LOF, missense		Missense	X	**>**
SETD2		LOF	Missense			**>**
TBL1XR1		LOF, missense				
UBAP2L		LOF			X	
UBR5			LOF	Missense	X	
BRCA1			LOF			
CNOT3		LOF			X	
ILF2		LOF				
NFIB		LOF				
CDC23		LOF				
NACC1		LOF			X	
SOX6		Missense				
ZNF219		Missense			X	
TRRAP		Missense 2 ×	Missense	Missense	X	
CHD4		Missense			X	
EP400			Missense		X	
NCOR1		Missense			X	
NR2F1			Missense		X	
ZHX3		Missense				
ZNF462			Missense		X	
KDM1A		Missense			X	
RUVBL1			Missense			
HDAC1		Missense				
MBD2			Missense			
CNOT1		Missense			X	
RCOR2		Missense				
SMC3			Missense		X	
TCF3			Missense			
ETV6		Missense				
ZEB1				LOF		
SMARCC2				LOF	X	
ANAPC5				Missense		
WIZ				Missense	X	
KDM3B				Missense	X	
ZFR				Missense		
MAML2				Missense		
QSER1				Missense		
RIF1				Missense		
NCOR2				Missense		
ZBTB45				Missense	X	
PBX1				Missense		

Abbreviations: ASD, autism spectrum disorder; ID, intellectual disability; IQ, intelligence quotient; LOF, loss of function; MD, mental disorder.

Human equivalent genes of network proteins with mutations in patients with the indicated mental disorders are listed. Predominant type of gene mutations, LOF or missense, in ID patients is listed. Type and number (if more than one) of gene mutations in patients with ASD-lowIQ, patients with ASD-normIQ or patients with schizophrenia are listed. Overlap with a list of 1003 constrained genes in the human population is indicated. Network proteins with a missense mutation in the human equivalent gene in patients with ASD-normIQ or schizophrenia and a LOF mutation in patients with ID or ASD-lowIQ are marked by >. The opposite pattern is not observed.
