# Supplementary figures and images for: An interaction network of mental disorder proteins in neural stem cells
Source: Transl Psychiatry. 2017 Apr 4;7(4):e1082–. doi: 10.1038/tp.2017.52 (PMC5416693; doi:10.1038/tp.2017.52)

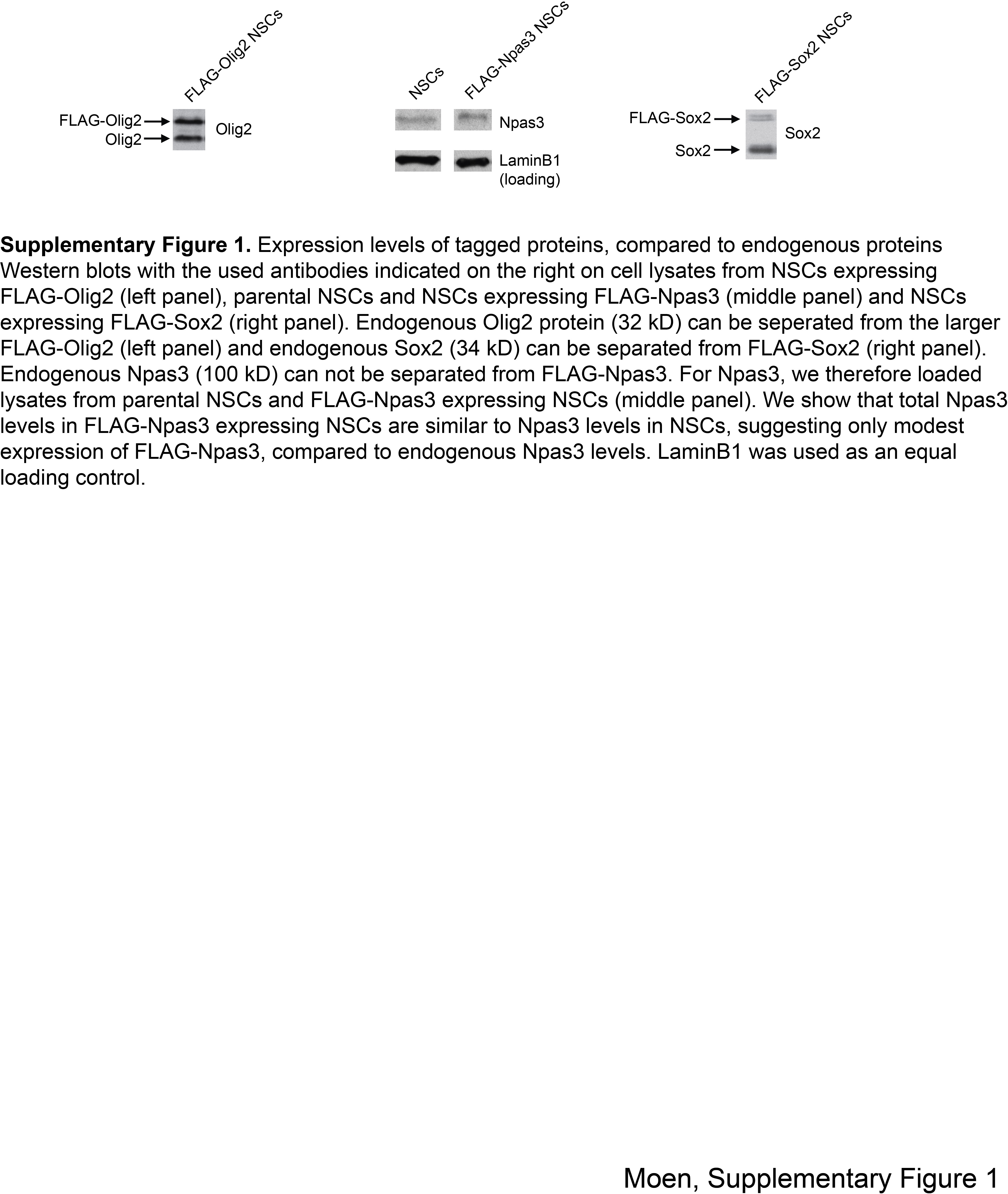

Supplement: Supplementary Figure 1 [file tp201752x1.tif]

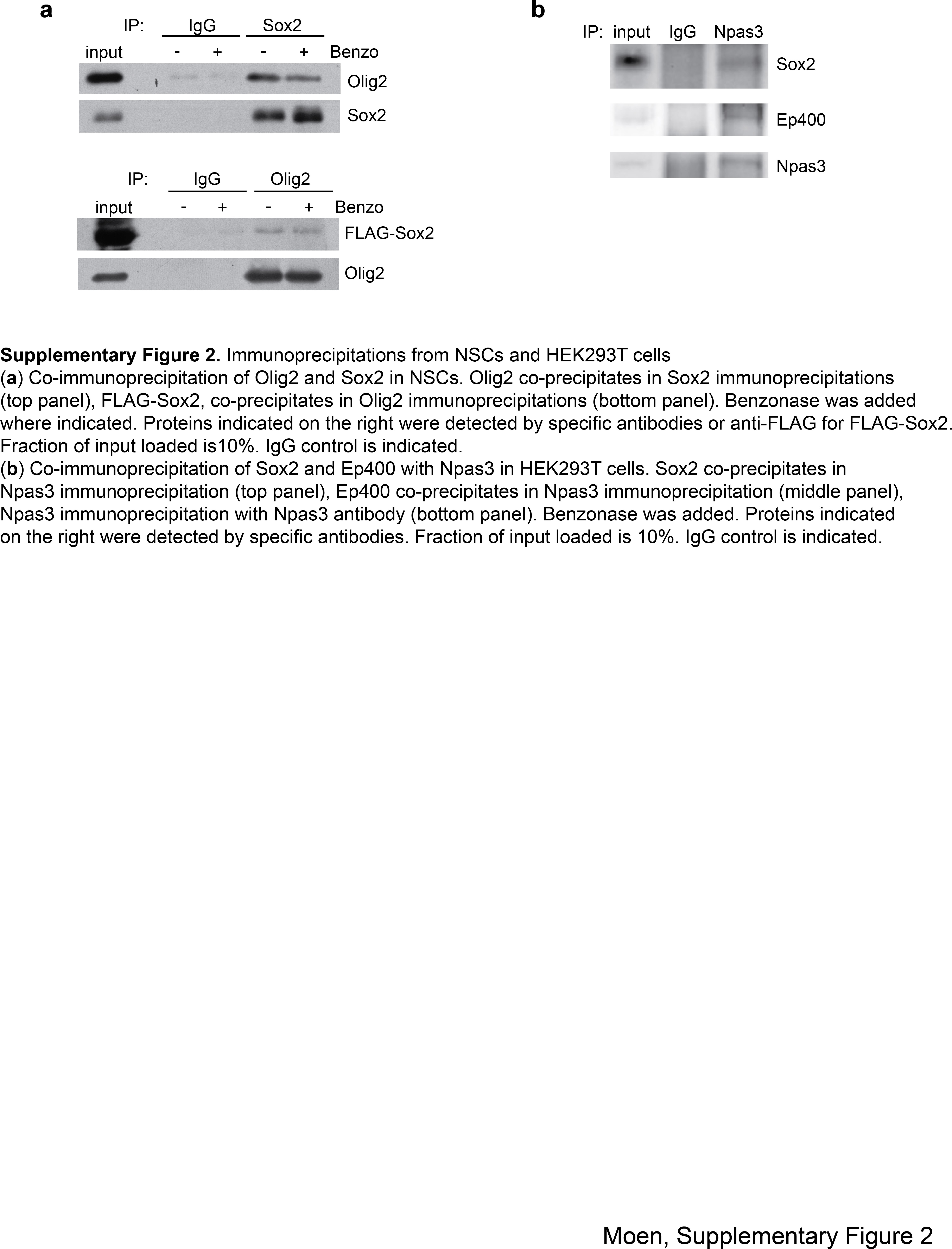

Supplement: Supplementary Figure 2 [file tp201752x2.tif]

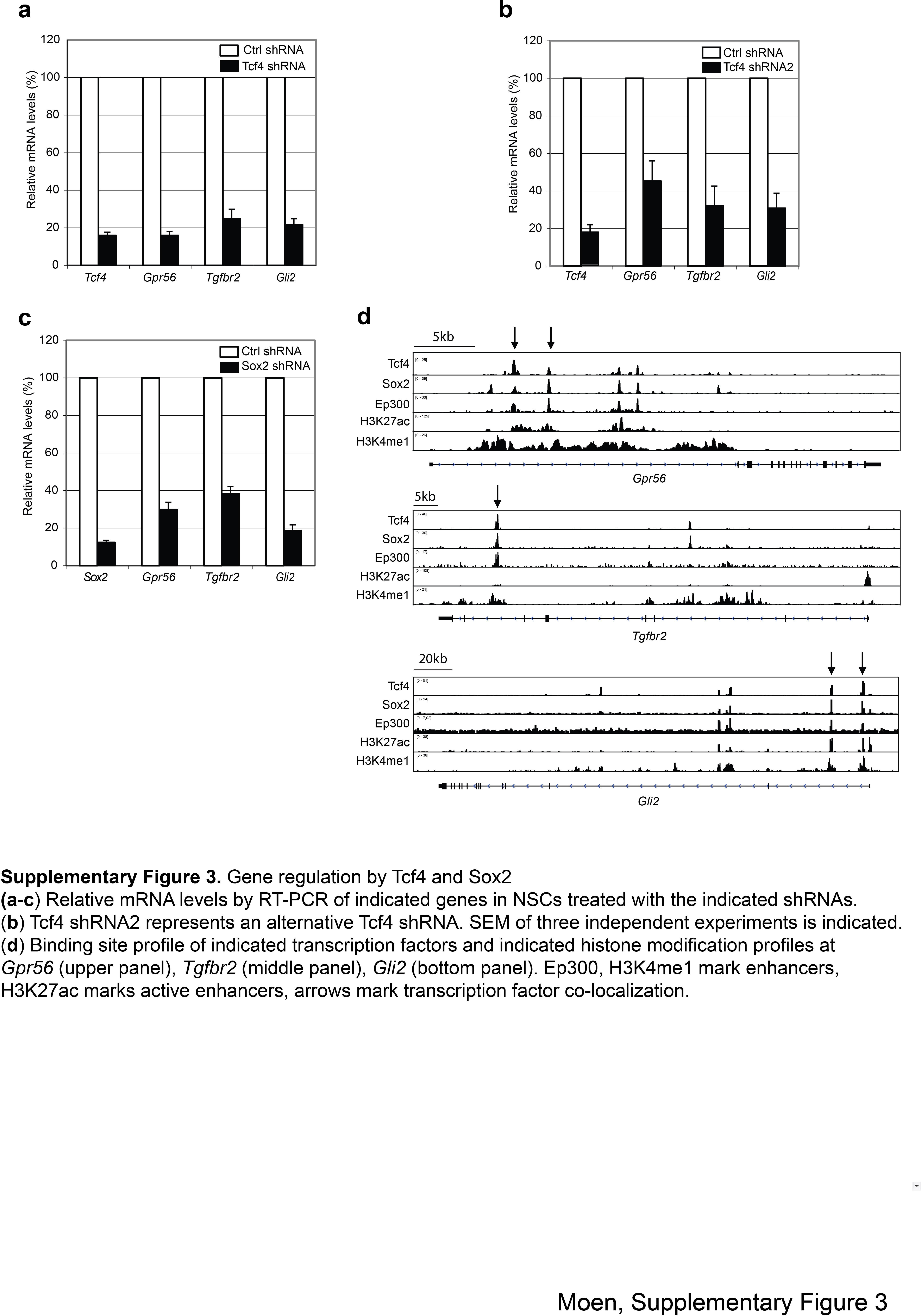

Supplement: Supplementary Figure 3 [file tp201752x3.tif]
