# Supplementary material for: An interaction network of mental disorder proteins in neural stem cells
Source: Transl Psychiatry. 2017 Apr 4;7(4):e1082–. doi: 10.1038/tp.2017.52 (PMC5416693; doi:10.1038/tp.2017.52)
Supplement: Supplementary Tables 1, 2, 3, 11 and Legends [file tp201752x4.docx]

**Supplementary Tables**

**Supplementary Tables 1 and 2**. Biological relevance of previously identified interactions with our purification protocol

Our purification protocol[^1^](#_ENREF_1) has been used by us[^1-7^](#_ENREF_1) and others[^8^](#_ENREF_8) to purify proteins and identify their interaction partners by mass spectrometry. The biological relevance of many of these identified protein-protein interactions was determined and is listed in Supplementary Table 1, if the biological relevance was determined by us (R. Poot is author), or listed in Supplementary Table 2 if the biological relevance was determined independently by others. Note that in Supplementary Table 2, all interactions, except the last one (Rnf12-Rex1), were identified by us but validation and determination of their biological relevance was performed independently by others.

**Supplementary Table 1.** Previously identified interactions by our purification protocol and their biological relevance, determined by us

| Interaction | Biological relevance | Reference |
| --- | --- | --- |
| Jarid2-PRC2 complex | Transcriptional priming of PRC2 target genes | [^6^](#_ENREF_6) |
| Pcl2-PRC2 complex | PRC2 recruitment to inactive X chromosome and PRC2 target genes | [^7^](#_ENREF_7) |
| Sox2-Chd7 | Sox2 and Chd7 cooperate in the regulation of common set of target genes, including disease-associated genes | [^5^](#_ENREF_5) |
| Rybp-PRC1 complex | PRC1 recruitment to target genes in an H3K27me3-independent pathway | [^4^](#_ENREF_4) |
| Nanog-Sox2 | Nanog-Sox2 interaction is important for ES cell self-renewal | [^3^](#_ENREF_3) |
| Pax6-Brg1 complex | Pax6-Brg1 complex regulates genes in adult neural stem cells to potentiate neurogenesis | [^2^](#_ENREF_2) |

**Supplementary Table 2.** Previously identified interactions by our purification protocol and their biological relevance, determined independently by others

| Interaction | Biological relevance | References |
| --- | --- | --- |
| Esrrb-Dax1 | Dax1 regulates the activity of Esrrb as a transcriptional activator | Biological relevance,[^9^](#_ENREF_9) interaction identification[^1^](#_ENREF_1)^,^ [^10^](#_ENREF_10) |
| Oct4-Sall4 | Sall4 stimulates transcriptional activation by Oct4 | Biological relevance,[^11^](#_ENREF_11) interaction identification[^1^](#_ENREF_1)^,^ [^10^](#_ENREF_10) |
| Oct4-Wdr5 | Oct4-directed Wdr5 binding to promoters of Oct4 target genes promotes the H3K4me3 mark and gene activation | Biological relevance,[^12^](#_ENREF_12) interaction identification[^1^](#_ENREF_1) |
| Esrrb-Ncoa3 | Ncoa3 physically links Esrrb to RNA polymerase 2 | Biological relevance,[^13^](#_ENREF_13) interaction identification[^1^](#_ENREF_1) |
| Oct4-Ogt | O-GlcNac modification of threonine 228 on Oct4 facilitates the activity of Oct4 in ESC maintenance and reprogramming to iPSCs | Biological relevance,[^14^](#_ENREF_14) interaction identification[^1^](#_ENREF_1)^,^ [^15^](#_ENREF_15) |
| Oct4-SWI-SNF-NuRD complex | Single amino acid changes in Oct4 reduce its binding to SWI-SNF and NuRD complex and abolish its activity in reprogramming to iPSCs | Biological relevance,[^16^](#_ENREF_16) interaction identification[^1^](#_ENREF_1)^,^ [^15^](#_ENREF_15) |
| Rnf12-Rex1 | Rnf12 targets Rex1 for degradation to allow for X-chromosome inactivation | Biological relevance and interaction identification[^8^](#_ENREF_8) |

**Supplementary Table 3.** Relative mRNA levels of indicated genes in used neural stem cells from our RNA-seq data

| Gene | Relative expression (rkpm)^a^ |
| --- | --- |
| *Tcf4* | 18.7 ± 0.5 |
| *Olig2* | 183.7 ± 11.2 |
| *Npas3* | 5.2 ± 0.3 |
| *Sox2* | 48.8 ± 3.2 |
| *Smad4* | 15.2 ± 0.3 |
| *Chd7* | 7.3 ± 0.1 |
| *Ascl1* | 8.3 ± 0.4 |
| *Ep300* | 7.8 ± 0.4 |
| *Brn2 (Pou3f2)* | 6.7 ± 2.0 |
| *Max* | 19.0 ± 0.3 |
| *Phox2b* | 0.1 ± 0.04 |
| Mean gene | 17.0 |
| Median gene | 0.8 |

^a^ SD of 3 independent RNA samples is indicated

**Supplementary Tables 4-7.** Interacting proteins of Tcf4, Olig2, Npas3 and Sox2, as identified by mass spectrometry analysis of purified protein samples (supplied as xlsx file with four tabs)

For each purified FLAG-tagged transcription factor, the names of the interacting proteins, accession numbers, emPAI scores (a relative and semi-quantitative measure of molar amount of protein,[^17^](#_ENREF_17) Mascot scores (a measure of correct protein identification) and number of identified unique peptides are indicated for independent experiments and a control experiment using parental NSCs. Proteins are categorized by their stable complex, or being a transcription factor (have a link to transcriptional regulation in the Uniprot protein database).

**Supplementary Table 8.** Network proteins and their overlap with different MD categories and constrained human genes (supplied as xlsx file)

Network proteins are alphabetically listed and overlaps with known ID genes[^18^](#_ENREF_18) or human genes *de novo* mutated in patients with ASD-lowIQ,[^19^](#_ENREF_19) ASD-normIQ^[19](#_ENREF_19)^ or schizophrenia[^20-23^](#_ENREF_20) are indicated. Overlap with a set of 1003 constrained human genes[^24^](#_ENREF_24) is indicated.

**Supplementary Table 9.** Network protein interactions and evidence of their biological relevance (supplied as xlsx file)

Interactions of bait proteins Tcf4, Olig2, Npas3 and Sox2 are listed in 4 subsequent tabs. Interactions between bait proteins are only listed once. Reference numbers for a biological function of the indicated interactions are in the third columns and references are listed below.[^5^](#_ENREF_5)^,^ [^25-35^](#_ENREF_25)

**Supplementary Table 10.** Genes with *de novo* mutations in schizophrenia patients from literature (supplied as xlsx file)

Human genes with *de novo* loss of function (LOF) and missense mutations were derived from the published studies.[^20-23^](#_ENREF_20) Genes are ordered alphabetically. Type of mutation and source publication are indicated. Frameshift mutations, nonsense mutations and splice site mutations (within 2 nucleotides from the splice donor site or splice acceptor site[^20^](#_ENREF_20)) were taken as LOF mutations. Genes appear more than once in the list if multiple mutations were identified.

**Supplementary Table 11.** Protein interactions between network factors associated with ID and network factors mutated in patients with ASD and/or schizophrenia

|  | Protein A | Protein B | MD protein A | MD protein B |
| --- | --- | --- | --- | --- |
| 1 | Tcf4 | Zeb2 | ID | ID, ASD-normIQ |
| 2 | Tcf4 | Chd7 | ID | ID, ASD-lowIQ |
| 3 | Tcf4 | Rfx3 | ID | ID, schizophrenia |
| 4 | Tcf4 | Nfia | ID | ID, ASD-lowIQ |
| 5 | Tcf4 | Ahdc1 | ID | ASD-lowIQ, schizophrenia |
| 6 | Tcf4 | Setd2 | ID | ASD-lowIQ, ASD-normIQ |
| 7 | Tcf4 | Tbl1xr1 | ID | ASD-lowIQ |
| 8 | Tcf4 | Ubap2l | ID | ASD-lowIQ |
| 9 | Tcf4 | Ubr5 | ID | ASD-normIQ |
| 10 | Tcf4 | Brca1 | ID | ASD-normIQ |
| 11 | Tcf4 | Ilf2 | ID | ASD-lowIQ |
| 12 | Tcf4 | Nfib | ID | ASD-lowIQ |
| 13 | Tcf4 | Cdc23 | ID | ASD-lowIQ |
| 14 | Tcf4 | Nacc1 | ID | ASD-lowIQ |
| 15 | Tcf4 | Trrap | ID | ASD-lowIQ, ASD-normIQ, schizophrenia |
| 16 | Tcf4 | Chd4 | ID | ASD-lowIQ |
| 17 | Tcf4 | Ep400 | ID | ASD-normIQ |
| 18 | Tcf4 | Ncor1 | ID | ASD-lowIQ |
| 19 | Tcf4 | Zfp462 | ID | ASD-normIQ |
| 20 | Tcf4 | Kdm1a | ID | ASD-lowIQ |
| 21 | Tcf4 | Mbd2 | ID | ASD-normIQ |
| 22 | Tcf4 | Cnot1 | ID | ASD-lowIQ |
| 23 | Tcf4 | Rcor2 | ID | ASD-lowIQ |
| 24 | Tcf4 | Tcf3 | ID | ASD-normIQ |
| 25 | Tcf4 | Zeb1 | ID | schizophrenia |
| 26 | Tcf4 | Anapc5 | ID | schizophrenia |
| 27 | Tcf4 | Wiz | ID | schizophrenia |
| 28 | Tcf4 | Zfr | ID | schizophrenia |
| 29 | Tcf4 | Maml2 | ID | schizophrenia |
| 30 | Tcf4 | Qser1 | ID | schizophrenia |
| 31 | Tcf4 | Rif1 | ID | schizophrenia |
| 32 | Tcf4 | Ncor2 | ID | schizophrenia |
| 33 | Tcf4 | Smarcc2 | ID | schizophrenia |
| 34 | Sox2 | Zeb2 | ID | ID, ASD-normIQ |
| 35 | Sox2 | Chd7 | ID | ID, ASD-lowIQ |
| 36 | Sox2 | Rfx3 | ID | ID, schizophrenia |
| 37 | Sox2 | Tbl1xr1 | ID | ASD-lowIQ |
| 38 | Sox2 | Nfib | ID | ASD-lowIQ |
| 39 | Sox2 | Nacc1 | ID | ASD-lowIQ |
| 40 | Sox2 | Trrap | ID | ASD-lowIQ, ASD-normIQ, schizophrenia |
| 41 | Sox2 | Chd4 | ID | ASD-lowIQ |
| 42 | Sox2 | Ruvbl1 | ID | ASD-normIQ |
| 43 | Sox2 | Hdac1 | ID | ASD-lowIQ |
| 44 | Sox2 | Cnot1 | ID | ASD-lowIQ |
| 45 | Sox2 | Zeb1 | ID | schizophrenia |
| 46 | Sox2 | Smarcc2 | ID | schizophrenia |
| 47 | Sox2 | Ncor2 | ID | schizophrenia |

Interacting network proteins (protein A and protein B) are listed and their overlap with known ID genes[^18^](#_ENREF_18) and/ or human genes *de novo* mutated in patients with ASD-lowIQ,[^19^](#_ENREF_19) ASD-normIQ^[19](#_ENREF_19)^ or schizophrenia[^20-23^](#_ENREF_20) are indicated.

**Supplementary Table 12.** Tcf4 target genes and Tcf4 binding sites (supplied as xlsx file)

First tab: Tcf4 target genes. Log2 of fold change and adjusted P-value of change in expression of target gene upon Tcf4 knock-down, total number of significant Tcf4 binding sites within 100 kb of the transcription start site (TSS) and distance of the nearest Tcf4 binding site (in basepairs) to the TSS are indicated. Second tab: Tcf4 binding sites in NSCs, determined by ChIP-seq, sorted by chromosome. Indicated are Chromosome (Chr), Start, end and summit of sequence read peak, the number of reads per peak and -10*log10(p-value), determined by MACS 1.4.2.

**Supplementary Table 13.** Tcf4 target gene overlap with genes associated with ID, ASD, schizophrenia or primary microcephaly (supplied as xlsx file)

Tcf4 target genes (Supplementary Table 12) overlapping with ID genes,[^18^](#_ENREF_18) genes *de novo* mutated in patients with ASD,[^19^](#_ENREF_19) genes *de novo* mutated in schizophrenia patients (Supplementary Table 10) or known primary microcephaly genes are indicated.

**Supplementary references**

1. van den Berg DL, Snoek T, Mullin NP, Yates A, Bezstarosti K, Demmers J *et al.* An Oct4-centered protein interaction network in embryonic stem cells. *Cell stem cell* 2010; **6**(4)**:** 369-381.

2. Ninkovic J, Steiner-Mezzadri A, Jawerka M, Akinci U, Masserdotti G, Petricca S *et al.* The BAF complex interacts with Pax6 in adult neural progenitors to establish a neurogenic cross-regulatory transcriptional network. *Cell stem cell* 2013; **13**(4)**:** 403-418.

3. Gagliardi A, Mullin NP, Ying Tan Z, Colby D, Kousa AI, Halbritter F *et al.* A direct physical interaction between Nanog and Sox2 regulates embryonic stem cell self-renewal. *EMBO J* 2013; **32**(16)**:** 2231-2247.

4. Tavares L, Dimitrova E, Oxley D, Webster J, Poot R, Demmers J *et al.* RYBP-PRC1 complexes mediate H2A ubiquitylation at polycomb target sites independently of PRC2 and H3K27me3. *Cell* 2012; **148**(4)**:** 664-678.

5. Engelen E, Akinci U, Bryne JC, Hou J, Gontan C, Moen M *et al.* Sox2 cooperates with Chd7 to regulate genes that are mutated in human syndromes. *Nat Genet* 2011; **43**(6)**:** 607-611.

6. Landeira D, Sauer S, Poot R, Dvorkina M, Mazzarella L, Jorgensen HF *et al.* Jarid2 is a PRC2 component in embryonic stem cells required for multi-lineage differentiation and recruitment of PRC1 and RNA Polymerase II to developmental regulators. *Nat Cell Biol* 2010; **12**(6)**:** 618-624.

7. Casanova M, Preissner T, Cerase A, Poot R, Yamada D, Li X *et al.* Polycomblike 2 facilitates the recruitment of PRC2 Polycomb group complexes to the inactive X chromosome and to target loci in embryonic stem cells. *Development* 2011; **138**(8)**:** 1471-1482.

8. Gontan C, Achame EM, Demmers J, Barakat TS, Rentmeester E, van IW *et al.* RNF12 initiates X-chromosome inactivation by targeting REX1 for degradation. *Nature* 2012; **485**(7398)**:** 386-390.

9. Uranishi K, Akagi T, Sun C, Koide H, Yokota T. Dax1 associates with Esrrb and regulates its function in embryonic stem cells. *Mol Cell Biol* 2013; **33**(10)**:** 2056-2066.

10. Wang J, Rao S, Chu J, Shen X, Levasseur DN, Theunissen TW *et al.* A protein interaction network for pluripotency of embryonic stem cells. *Nature* 2006; **444**(7117)**:** 364-368.

11. Tanimura N, Saito M, Ebisuya M, Nishida E, Ishikawa F. Stemness-related factor Sall4 interacts with transcription factors Oct-3/4 and Sox2 and occupies Oct-Sox elements in mouse embryonic stem cells. *J Biol Chem* 2013; **288**(7)**:** 5027-5038.

12. Ang YS, Tsai SY, Lee DF, Monk J, Su J, Ratnakumar K *et al.* Wdr5 mediates self-renewal and reprogramming via the embryonic stem cell core transcriptional network. *Cell* 2011; **145**(2)**:** 183-197.

13. Percharde M, Lavial F, Ng JH, Kumar V, Tomaz RA, Martin N *et al.* Ncoa3 functions as an essential Esrrb coactivator to sustain embryonic stem cell self-renewal and reprogramming. *Genes Dev* 2012; **26**(20)**:** 2286-2298.

14. Jang H, Kim TW, Yoon S, Choi SY, Kang TW, Kim SY *et al.* O-GlcNAc regulates pluripotency and reprogramming by directly acting on core components of the pluripotency network. *Cell Stem Cell* 2012; **11**(1)**:** 62-74.

15. Pardo M, Lang B, Yu L, Prosser H, Bradley A, Babu MM *et al.* An expanded Oct4 interaction network: implications for stem cell biology, development, and disease. *Cell stem cell* 2010; **6**(4)**:** 382-395.

16. Esch D, Vahokoski J, Groves MR, Pogenberg V, Cojocaru V, Vom Bruch H *et al.* A unique Oct4 interface is crucial for reprogramming to pluripotency. *Nat Cell Biol* 2013; **15**(3)**:** 295-301.

17. Ishihama Y, Oda Y, Tabata T, Sato T, Nagasu T, Rappsilber J *et al.* Exponentially modified protein abundance index (emPAI) for estimation of absolute protein amount in proteomics by the number of sequenced peptides per protein. *Mol Cell Proteomics* 2005; **4**(9)**:** 1265-1272.

18. Gilissen C, Hehir-Kwa JY, Thung DT, van de Vorst M, van Bon BW, Willemsen MH *et al.* Genome sequencing identifies major causes of severe intellectual disability. *Nature* 2014; **511**(7509)**:** 344-347.

19. Iossifov I, O'Roak BJ, Sanders SJ, Ronemus M, Krumm N, Levy D *et al.* The contribution of de novo coding mutations to autism spectrum disorder. *Nature* 2014; **515**(7526)**:** 216-221.

20. Fromer M, Pocklington AJ, Kavanagh DH, Williams HJ, Dwyer S, Gormley P *et al.* De novo mutations in schizophrenia implicate synaptic networks. *Nature* 2014; **506**(7487)**:** 179-184.

21. Xu B, Ionita-Laza I, Roos JL, Boone B, Woodrick S, Sun Y *et al.* De novo gene mutations highlight patterns of genetic and neural complexity in schizophrenia. *Nat Genet* 2012; **44**(12)**:** 1365-1369.

22. Gulsuner S, Walsh T, Watts AC, Lee MK, Thornton AM, Casadei S *et al.* Spatial and temporal mapping of de novo mutations in schizophrenia to a fetal prefrontal cortical network. *Cell* 2013; **154**(3)**:** 518-529.

23. Guipponi M, Santoni FA, Setola V, Gehrig C, Rotharmel M, Cuenca M *et al.* Exome sequencing in 53 sporadic cases of schizophrenia identifies 18 putative candidate genes. *PLoS ONE* 2014; **9**(11)**:** e112745.

24. Samocha KE, Robinson EB, Sanders SJ, Stevens C, Sabo A, McGrath LM *et al.* A framework for the interpretation of de novo mutation in human disease. *Nat Genet* 2014; **46**(9)**:** 944-950.

25. Einarson MB, Chao MV. Regulation of Id1 and its association with basic helix-loop-helix proteins during nerve growth factor-induced differentiation of PC12 cells. *Mol Cell Biol* 1995; **15**(8)**:** 4175-4183.

26. Langlands K, Yin X, Anand G, Prochownik EV. Differential interactions of Id proteins with basic-helix-loop-helix transcription factors. *J Biol Chem* 1997; **272**(32)**:** 19785-19793.

27. Persson P, Jogi A, Grynfeld A, Pahlman S, Axelson H. HASH-1 and E2-2 are expressed in human neuroblastoma cells and form a functional complex. *Biochem Biophys Res Commun* 2000; **274**(1)**:** 22-31.

28. Bai X, Kim J, Yang Z, Jurynec MJ, Akie TE, Lee J *et al.* TIF1gamma controls erythroid cell fate by regulating transcription elongation. *Cell* 2010; **142**(1)**:** 133-143.

29. Hsu HL, Huang L, Tsan JT, Funk W, Wright WE, Hu JS *et al.* Preferred sequences for DNA recognition by the TAL1 helix-loop-helix proteins. *Mol Cell Biol* 1994; **14**(2)**:** 1256-1265.

30. Wu D, Su X, Potluri N, Kim Y, Rastinejad F. NPAS1-ARNT and NPAS3-ARNT crystal structures implicate the bHLH-PAS family as multi-ligand binding transcription factors. *Elife* 2016; **5**.

31. Baltus GA, Kowalski MP, Tutter AV, Kadam S. A positive regulatory role for the mSin3A-HDAC complex in pluripotency through Nanog and Sox2. *J Biol Chem* 2009; **284**(11)**:** 6998-7006.

32. Wang S, Xia P, Ye B, Huang G, Liu J, Fan Z. Transient activation of autophagy via Sox2-mediated suppression of mTOR is an important early step in reprogramming to pluripotency. *Cell stem cell* 2013; **13**(5)**:** 617-625.

33. Gontan C, Guttler T, Engelen E, Demmers J, Fornerod M, Grosveld FG *et al.* Exportin 4 mediates a novel nuclear import pathway for Sox family transcription factors. *J Cell Biol* 2009; **185**(1)**:** 27-34.

34. Chen B, Lim RW. Physical and functional interactions between the transcriptional inhibitors Id3 and ITF-2b. Evidence toward a novel mechanism regulating muscle-specific gene expression. *J Biol Chem* 1997; **272**(4)**:** 2459-2463.

35. Goldfarb AN, Lewandowska K, Shoham M. Determinants of helix-loop-helix dimerization affinity. Random mutational analysis of SCL/tal. *J Biol Chem* 1996; **271**(5)**:** 2683-2688.
